# Takayasu Arteritis with Dyslipidemia Increases Risk of Aneurysm

**DOI:** 10.1038/s41598-019-50527-z

**Published:** 2019-10-01

**Authors:** Lili Pan, Juan Du, Dong Chen, Yanli Zhao, Xi Guo, Guanming Qi, Tian Wang, Jie Du

**Affiliations:** 1 Department of Rheumatology and Immunology, Beijing Anzhen Hospital, Capital Medical University, Beijing, China; 2 Department of Pathology, Beijing Anzhen Hospital, Capital Medical University, Beijing, China; 3 Department of Interventional Radiology, Beijing Anzhen Hospital, Capital Medical University, Beijing, China; 4 Pulmonary and Critical Care Division, Tufts Medical Center, Boston, MA USA; 5 Beijing Institute of Heart, Lung and Blood Vessel Disease, Beijing, China; 6 Beijing Anzhen Hospital, Capital Medical University, Key Laboratory of Remodeling-Related Cardiovascular Diseases, Ministry of Education, Beijing Collaborative Innovation Center for Cardiovascular Disorders, Beijing, China

**Keywords:** Vasculitis, Vasculitis

## Abstract

Low-density lipoprotein cholesterol (LDL-C) has been associated with the occurrence of abdominal aortic aneurysm. However, whether LDL-C elevation associated with aneurysms in large vessel vasculitis is unknown. The aim of this study is to investigate the clinical and laboratory features of Takayasu arteritis (TAK) and explore the risk factors that associated with aneurysm in these patients. This retrospective study compared the clinical manifestations, laboratory parameters, and imaging results of 103 TAK patients with or without aneurysms and analyzed the risk factors of aneurysm formation. 20.4% of TAK patients were found to have aneurysms. The LDL-C levels was higher in the aneurysm group than in the non-aneurysm group (2.9 ± 0.9 mmol/l vs. 2.4 ± 0.9 mmol/l, p = 0.032). Elevated serum LDL-C levels increased the risk of aneurysm by 5.8-fold (p = 0.021, odds ratio [OR] = 5.767, 95% confidence interval [CI]: 1.302–25.543), and the cutoff value of level of serum LDL-C was 3.08 mmol/l. The risk of aneurysm was 4.2-fold higher in patients with disease duration >5 years (p = 0.042, OR = 4.237, 95% CI: 1.055–17.023), and 2.9-fold higher when an elevated erythrocyte sedimentation rate was present (p = 0.077, OR = 2.851, 95% CI: 0.891–9.115). In this study, elevated LDL-C levels increased the risk of developing aneurysms in patients with TAK.

## Introduction

Aneurysmal disease is a life-threatening condition which frequently affects large elastic arteries such as the aorta. Previously, it was considered a “degenerative” condition. The risk factors of development of abdominal aortic aneurysms (AAA) include advanced age, male sex, smoking, hypertension, atherosclerosis and peripheral vascular disease^1,2^. Recently, a 24 year prospective study revealed that elevated low-density lipoprotein cholesterol (LDL-C) level did not only increased the future risk of clinical AAA, but was also associated with asymptomatic AAA^3^. Another genetic study confirmed the association of elevated LDL-C with AAA risk, and indicated that lowering LDL-C levels could be an effective therapeutic approach for prevention and management of AAA^4^. However, AAA is not associated with any single gene mutation or genetic locus, suggesting that it is a complex disorder, and so, management may require consideration of several factors. Enhanced magnetic resonance imaging (MRI) with the use of ultra-small superparamagnetic iron oxide particles has demonstrated that aortic wall inflammation can predict the rate of aneurysm growth and the risk of aneurysm rupture or repair, as well as all-cause and aneurysm-related mortality^5^. The influence of large vessel vasculitis on the incidence of aneurysm formation is unclear in patients with traditional cardiovascular risk factors.

Takayasu arteritis (TAK) is an autoimmune, large-vessel vasculitis that is mainly involved in the aorta and its major branches. It has unknown etiology and typically affects Asian women under the age of 40 years. Chronic inflammation may induce intimal hyperplasia and fibrosis of the media and intima, resulting in luminal stenosis and occlusion in more than 90% of patients with TAK. The formation of aneurysms is not rare in TAK patients, a multicenter study in France indicating the prevalence of aneurysms in such patients is 24%^6^. Although TAK is typically characterized by stenotic lesions, autopsy-based research has shown that over 50% of individuals with TAK may also develop aneurysms^7^. Aneurysmal lesions occur from the aortic root to the arch at a higher frequency in Asian people than in Western people^8^, and are one of the main causes of death in Chinese TAK patients^9^. There is a lack of knowledge about aneurysm risk in TAK patients due to the rarity of the disease. Previous studies have found that prevalent of dyslipidemia in patients with TAK was 19–47%^10–13^, and that lipids disorders are related to the activity of TAK^14^. The purpose of this study was to investigate the clinical and laboratory features of TAK and to analyze the combination of TAK and dyslipidemia as a risk factor for aneurysm formation.

## Methods

### Ethics

This retrospective study was conducted in accordance with the ethical principles of the Declaration of Helsinki and approved by the Ethics Committee of Beijing Anzhen Hospital (approval number:2018013X), Capital Medical University. All experiments were performed in accordance with relevant named guidelines and regulations and consent was obtained from all participants and/or their legal guardians.

### Participants

This retrospective cross-sectional study recruited 103 consecutive patients with TAK from the Department of Rheumatology and Immunology, Beijing Anzhen Hospital, from January 2012 to December 2017. Inclusion criteria was decided according to the criteria for classification of TAK developed by the American College of Rheumatology in 1990^15^. Exclusion criteria was: patients with other autoimmune diseases, cancer, or infection. Disease activity was assessed using a modified version of Kerr’s criteria^16^ and the Indian Takayasu Clinical Activity Score (ITAS)^17^. Patients were divided into two groups according to the presence or absence of aneurysm (the aneurysm and non-aneurysm groups).

We retrospectively reviewed the patients’ baseline general information, medical history, clinical manifestations, laboratory parameters, and angiographic findings. We defined dyslipidemia as range of lipid abnormalities and may involve a combination of increased total cholesterol (>5.20 mmol/l), LDL-C(>3.12 mmol/l), and triglyceride levels (>1.7 mmol/l) or decreased HDL-C (<1.04 mmol/l). Hypertension was defined as a systolic blood pressure ≥140 mmHg and/or a diastolic blood pressure ≥90 mmHg and/or use of blood pressure lowering medication. We obtained information of aorta and its branches using magnetic resonance angiography (MRA), computed tomography angiography (CTA) or Doppler ultrasound. Heart failure defined as left ventricular EF <40% by echocardiogram.

### Angiographic classification and features

Aneurysm was defined as arterial dilation to more than 50% of the normal diameter of the artery^18^. The MRA was used to evaluate the thoracic aorta and its branches, CTA or Doppler ultrasound to measure the abdominal aorta and its branches and peripheral arteries. Lesions were classified according to the angiographic classification of the 1994 International TAK Conference in Tokyo^19^. The distribution of lesions was classified as follows: Numano type I (branches of the aortic arch), type IIa (ascending aorta, aortic arch, and its branches), type IIb (ascending aorta, aortic arch and its branches, and thoracic descending aorta), type III (thoracic descending aorta, abdominal aorta, and/or renal arteries), type IV (abdominal aorta and/or renal arteries), and type V (combined features of types IIb and IV).

### Collection of blood samples

For each subject, 4 mL of venous blood was drawn in the morning after a 12-hour fasting period. Blood was placed into a tube without anticoagulant. After the blood coagulated, it was centrifuged at 3,000 r/m for 5 minutes, and the serum was collected. A Hitachi 7600–120 automatic biochemical analyzer (Tokyo, Japan) was used to analyze serum parameters. The ESR was measured using the modified Westergren method in a standardized manner.

### Pathological staining of aortic tissue

Specimens were fixed in 4% neutral formalin for 24 hours, embedded in paraffin, sectioned, and stained with hematoxylin and eosin. Masson stains and Verhoeff-van Gieson elastic stains were also evaluated to demonstrate areas of degeneration, elastic fiber disorder and fragmentation, and accumulation of collagen, proteoglycans and mucopolysaccharids.

### Statistical analysis

Values are expressed as the mean ± standard error. Differences between measured parameters in the two groups were assessed using an unpaired *t* test. When data were not normally distributed, the Mann-Whitney U test was used, and these values are expressed as quartiles. Qualitative parameters were assessed using the χ^2^ test. All statistical tests were two-tailed, and p-values < 0.05 were considered to indicate statistical significance. To investigate the potential risk factors of aneurysm, following variables were included the in the logistic regression model: age at disease onset (years) (≤19 = 1, 20–39 = 2, 40–59 = 3, ≥60 = 4), male gender, disease duration (months) (≤60 = 0, >60 = 1), fever, chest pain, arteriosclerosis, hypertension, serum total cholesterol (TC), LDL-C levels, C-reactive protein (CRP) levels, ESR, Kerr’s Score, ITAS and treatment with glucocorticoids (GCs). We calculated the cutoff values of TC and LDL-C by using ROC curve. Backward stepwise regression was used with odds ratios (ORs) and the corresponding 95% confidence intervals (CIs) in the model (p = 0.05 entry and p = 0.10 removal criteria), p-values < 0.05 were considered to indicate statistical significance. All statistical analyses were performed using SPSS 20.0 statistical software (SPSS Inc., Chicago, IL, USA).

## Results

### The demographic and clinical features of Takayasu Arteritis patients with and without aneurysms

Among the 103 hospitalized TAK patients, 20.4% (21/103) suffer aneurysms. Among them, 15 were women and 6 were men (2.5:1), the mean age of TAK onset was 23.4 years, and the median duration of disease was 96 months. There was no significant difference in age, sex, disease duration, and body mass index at disease onset in TAK patients with or without aneurysms. We compared the history of hypertension, dyslipidemia, coronary artery disease, type 2 diabetes mellitus, arteriosclerosis, smoking, heart failure, stroke or transitory ischemic attack, and history of tuberculosis between the aneurysm and non-aneurysm group. No significant difference was observed between the two groups in these factors (Table 1).

**Table 1:** Clinical Features of TA patients with or without aneurysm

	Aneurysm (n = 21)	Non-aneurysm (n = 82)	P-value
Age of onset (years)	23.4 ± 11.2	37.2 ± 12.2	0.096
Gender (female) n(%)	15 (71.4)	71 (86.6)	0.180
Disease duration (months)	96 (6.5, 174.0)	36 (2.35, 155.5)	0.249
BMI (kg/m^2^)	23.1 ± 3.9	22.3 ± 3.5	0.330
Hypertension, n (%)	9 (42.9)	33 (40.2)	0.828
Dyslipidemia, n (%)	12(57.1)	28 (34.1)	0.054
Coronary artery disease, n (%)	0 (0.0)	6 (7.3)	0.450
T2DM, n (%)	0 (0.0)	4 (4.9)	0.304
Arteriosclerosis, n(%)	10 (47.6)	35 (42.7)	0.684
Smoker, n (%)	5 (23.8)	10 (12.2)	0.317
Stroke/TIA, n (%)	0 (0.0)	3 (3.7)	0.376
Heart failure, n (%)	6(28.6)	10(12.2)	0.131
history of TB, n (%)	4 (19.0)	13 (15.9)	0.982
Dizziness, n (%)	8 (38.1)	44 (53.7)	0.203
Headache, n (%)	2 (9.5)	15 (18.3)	0.524
Pulseless, n (%)	4 (19.0)	23 (28.1)	0.403
Asymmetry in BP, n (%)	7 (33.3)	31 (37.8)	0.705
Claudication, n (%)	2 (9.5)	12 (14.6)	0.800
Fever, n (%)	7 (33.3)	10 (12.2)	0.046
Palpitations, n (%)	5 (23.8)	8 (9.8)	0.173
Chest tightness, n (%)	4 (19.0)	23 (28.1)	0.403
Chest pian, n (%)	9 (42.9)	14 (17.1)	0.025
Abdominal pain, n (%)	0 (0.0)	0 (0.0)	—
Carotidynia, n (%)	2 (9.5)	9 (11.0)	1.000
Erythema nodosum, n (%)	1 (4.8)	1 (1.2)	0.296
Weight loss, n (%)	1 (4.8)	5 (6.1)	1.000
**Treatments**, **n** (**%)**			
Corticosteroids	15 (71.4)	58 (70.7)	0.950
CTX	9 (42.9)	47 (57.3)	0.235
MTX	5 (23.8)	11 (13.4)	0.403
MMF	4 (19.0)	9 (11.0)	0.532
LEF	1 (4.8)	5 (6.1)	1.000
HCQ	1 (4.8)	8 (9.8)	0.772
Tocilizumab	6 (28.6)	16 (19.5)	0.366
Statins	5 (23.8)	19 (23.2)	0.951

Note: BMI, Body mass index; TB, tuberculosis; TIA, Transitory ischemic attack; CTX, cyclophosphamide; MTX, methotrexate; MMF, mycophenolate mofetil; LEF, leflunomide; HCQ, hydroxychloroquine.

Regarding clinical manifestations, 33.3% (7/21) of TAK patients with aneurysms had a fever, which was a significantly higher proportion than the patients without aneurysms (12.2%, 10/82, p = 0.046). The incidence of chest pain in patients with aneurysms (42.9%, 9/21) was significantly higher than that of non-aneurysm group (17.1%, 14/82, p = 0.025). There were no significant difference in the incidence of dizziness, headache, pulseless, asymmetry in blood pressure (>10 mmHg), claudication, palpitations, chest tightness (described as “band-like,” “heavy weight” or “really tight” feelings), abdominal pain, carotidynia, erythema nodosum, or weight loss between the two groups. Over 70% of the patients in both groups were treated with GCs. Cyclophosphamide was the most frequently used immunosuppressant, followed by methotrexate. There was no significant difference in the use of disease-modifying anti-rheumatic drugs and cholesterol-lowering drugs between the two groups (Table 1).

### Angiographic manifestation of the affected arteries

**Figure 1:**
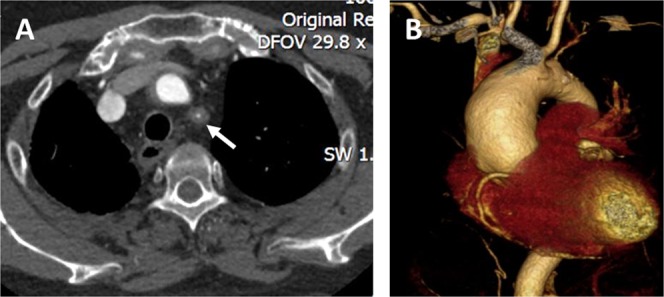
Computed tomography angiography of the aorta of a Takayasu arteritis patient. A 50-year-old female patient was diagnosed with Takayasu arteritis for 20 years. (**A**) Obvious thickening of the vascular wall leading to severe stenosis in the left subclavian artery (white arrow). (**B**) Aneurysm formation in the ascending aorta.

21 patients out of 103 TAK patients were found to have aneurysms, because some patients had multiple aneurysms, 28 aneurysms in total were found in these patients. Aortic aneurysms were found in 67.9% (19/28) of these patients: 16 thoracic aortic aneurysms (TAAs) and 3 AAAs (Fig. 1). The aneurysms were located in the ascending aorta in ten cases, the root of the aorta in two, the aortic arch in two, and the descending aorta in two. Peripheral artery aneurysms occurred in 32.1% (9/28) of aneurysms (in the subclavian artery in four cases, the iliac artery in two, the common carotid artery in one, the innominate artery in one, and the superior mesenteric artery in one). Among the 28 aneurysms, 26 were true aneurysms (92.9%), one was a dissecting aneurysm (abdominal aorta), and one was an anastomotic pseudoaneurysm (aortic arch) (Table 2). Of the 21 patients, sixteen had single aneurysms and five had multiple aneurysms.

**Table 2:** Location and nature of aneurysms in Takayasu arteritis patients.

	Number	%
**Location of aneurysm**		
**Thoracic aorta**	**16**	**57**.**1%**
root of the aorta	2	7.1%
ascending aorta	10	35.7%
aortic arch	2	7.1%
descending aorta	2	7.1%
**Abdominal aorta**	**3**	**10**.**7%**
**Peripheral aneurysm**	**9**	**32**.**1%**
subclavian artery	4	14.3%
iliac artery	2	7.1%
common carotid artery	1	3.6%
innominate artery	1	3.6%
superior mesenteric artery	1	3.6%
**Nature of aneurysm**		
true aneurysm	26	92.9%
dissecting aneurysm	1	3.6%
pseudoaneurysm	1	3.6%

Among the aneurysm group, Numano type V (57.1%, 12/21) was the most common angiographic type, followed by types IIa (14.3%, 3/21), IIb, and III (9.5% each [2/21]). The most prevalent angiographic type in the non-aneurysm group was Numano type V (42.7%, 35/82), followed by types I (25.6%, 21/82), IIb (20.7%, 17/82), III (4.9%, 4/82), and IIa (3.7%, 3/82). There was no significant difference in angiographic type between aneurysm and non-aneurysm groups.

### Comparison of laboratory parameters and disease activity in aneurysm and non-aneurysm takayasu arteritis patients

**Figure 2:**
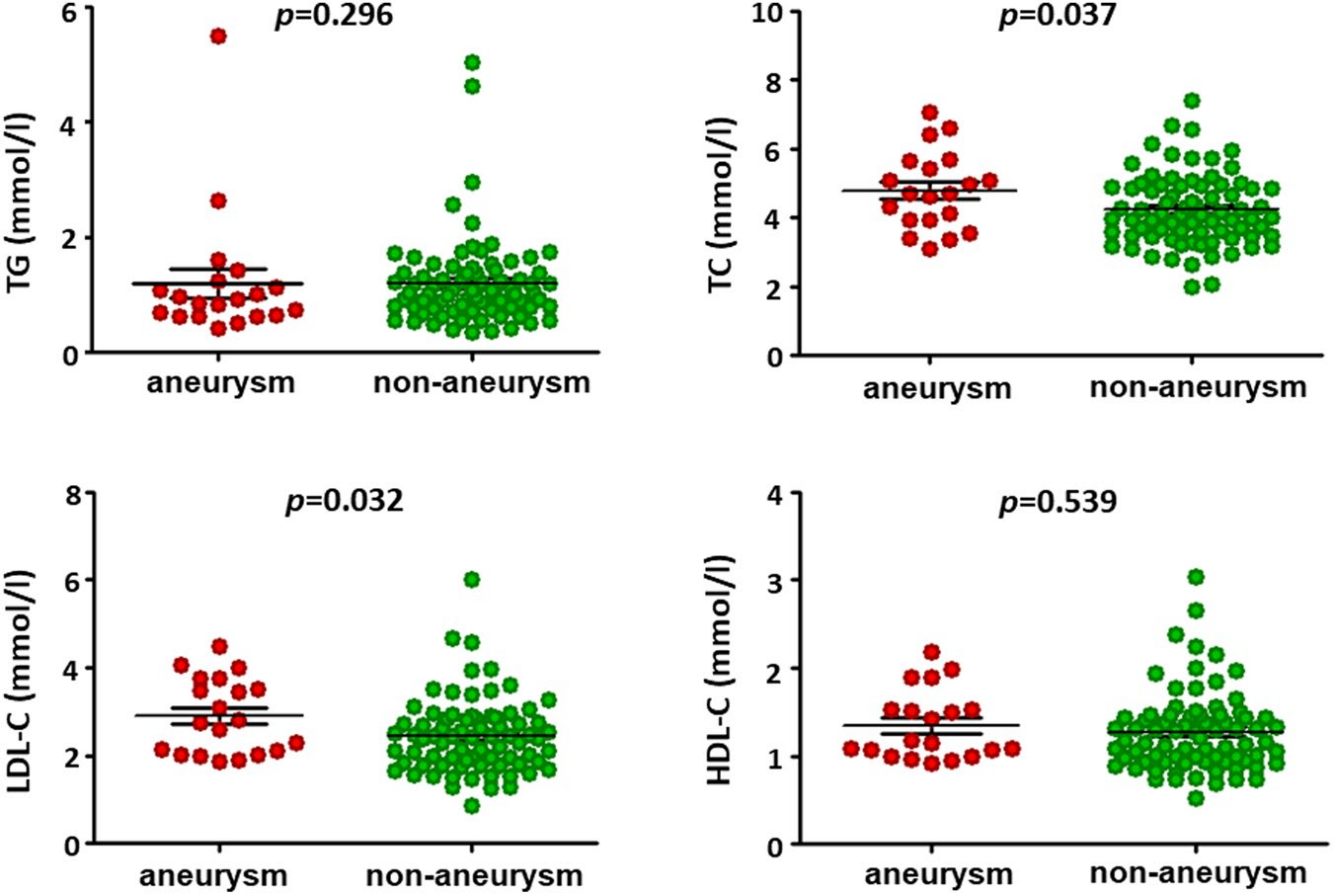
Levels of serum lipids in Takayasu arteritis patients with and without aneurysm. Serum level of total cholesterol and low density lipoprotein cholesterol in aneurysm group was significantly higher than that of patients without aneurysm (4.8 ± 1.1 vs 4.2 ± 1.0, p = 0.037) (2.9 ± 0.9 vs 2.4 ± 0.9, p = 0.032). No significant differences were found in level of triglyceride and high density lipoprotein cholesterol (HDL-C) in two groups.

The TC and LDL-C levels of patients in the aneurysm group were significantly higher than those in the non-aneurysm group (4.8 ± 1.1 mmol/l vs. 4.2 ± 1.0 mmol/l, p = 0.037; and 2.9 ± 0.9 mmol/l vs. 2.4 ± 0.9 mmol/l, p = 0.032, respectively). No significant differences were observed between the groups in the triglyceride (TG) levels (0.9 [0.6, 1.2] mmol/l vs 1.0 [0.8, 1.4] mmol/l, p = 0.296) or high-density lipoprotein cholesterol (HDL-C) levels (1.3 ± 0.4 mmol/l vs. 1.3 ± 0.6 mmol/l, p = 0.539) (Fig. 2). Immunity and inflammation markers such as serum tumor necrosis factor (TNF)-α, interleukin (IL)-6, immunoglobulin (Ig)A, IgG, IgM, IgE, complement 3, complement 4, and positive antinuclear antibodies were not significantly different between the two groups (Table 3).

**Table 3:** Laboratory parameters and disease activity in Takayasu arteritis patients with and without aneurysm.

	Aneurysm (n = 21)	Non-aneurysm (n = 82)	P-value
WBC (109/L)	8.6 ± 3.4	7.5 ± 2.2	0.096
NE (109/L)	5.6 ± 3.1	5.00 ± 1.9	0.262
LY (109/L)	2.2 (1.7, 2.9)	1.9 (1.5,2.4)	0.134
PLT (109/L)	250.1 ± 70.9	268.8 ± 89.	0.378
RBC (1012/L)	4.4 ± 0.5	4.5 ± 0.5	0.497
Hb (g/L)	125.8 ± 20.7	123.5 ± 18.7	0.619
ALT (U/L)	16.8 ± 14.6	17.9 ± 13.1	0.734
Cr (μmol/l)	58.4 ± 14.8	61.1 ± 27.6	0.666
Fasting glucose (mmol/l)	4.7 ± 0.5	5.0 ± 1.1	0.248
HbA1c (%)	5.8 ± 1.0	6.5 ± 1.9	0.426
HCY (μmol/l)	13.4 ± 8.1	13.46 ± 8.5	0.981
BNP (pg/ml)	92 (31.5, 421.5)	51.5 (19.8, 193.8)	0.462
TNI (ng/ml)	0.00 (0.0, 0.0)	0.00 (0.0, 0.0)	0.648
IL-6 (pg/ml)	6.6 (4.0, 18.5)	6.2 (2.2, 15.6)	0.412
TNF-α (pg/ml)	19.1 (6.2, 44.2)	14.1 (7.2, 27.6)	0.386
IgA (g/l)	2.3 ± 0.9	2.7 ± 1.4	0.274
IgG (g/l)	12.1 ± 5.0	12.9 ± 4.00	0.489
IgM (g/l)	1.7 ± 1.00	1.5 ± 1.4	0.674
IgE (g/l)	18.9 (9.7, 99.3)	19.0 (11.00, 56.1)	0.918
C3 (g/l)	1.2 ± 0.2	1.2 ± 0.3	0.548
C4 (g/l)	0.2 ± 0.1	0.2 ± 0.1	0.630
ANA(+) n,%	1 (4.8)	9 (11.0)	0.656
ESR(mm/1 h)	21.0 (3.0, 51.5)	17.0 (7.0,29.3)	0.918
CRP (mg/l)	5.0 (0.9,2 8.9)	2.9 (0.5, 17.3)	0.322
Kerr’s Score	2.4 ± 0.8	2.2 ± 0.8	0.323
ITAS	7.2 ± 3.6	6.3 ± 3.9	0.324

Note: WBC, white blood cell; LY, lymphocyte; NE, neutrophil; PLT, platelet; RBC, red blood cell; Hb, hemoglobin; ALT, alanine aminotransferase; Cr, creatinine; HbA1c, glycosylated hemoglobin; TG, triglyceride; TC, total cholesterol; LDL-C, low-density lipoprotein cholesterol; HDL-C, high-density lipoprotein cholesterol; HCY, homocysteine BNP, brain natriuretic peptide; TNI, troponin I; IL, interleukin; TNF, tumor necrosis factor; Ig, immunoglobulin; C3, complement 3; C4, complement 4; ESR, erythrocyte sedimentation rate; CRP, C-reactive protein.

In comparing the disease activity between the two groups, no statistically significant difference was found in ESR, CRP level, Kerr’s score, or ITAS between the aneurysm and non-aneurysm group (Table 3).

### Histopathological features of the aortic wall in a patient with takayasu arteritis aneurysm

**Figure 3:**
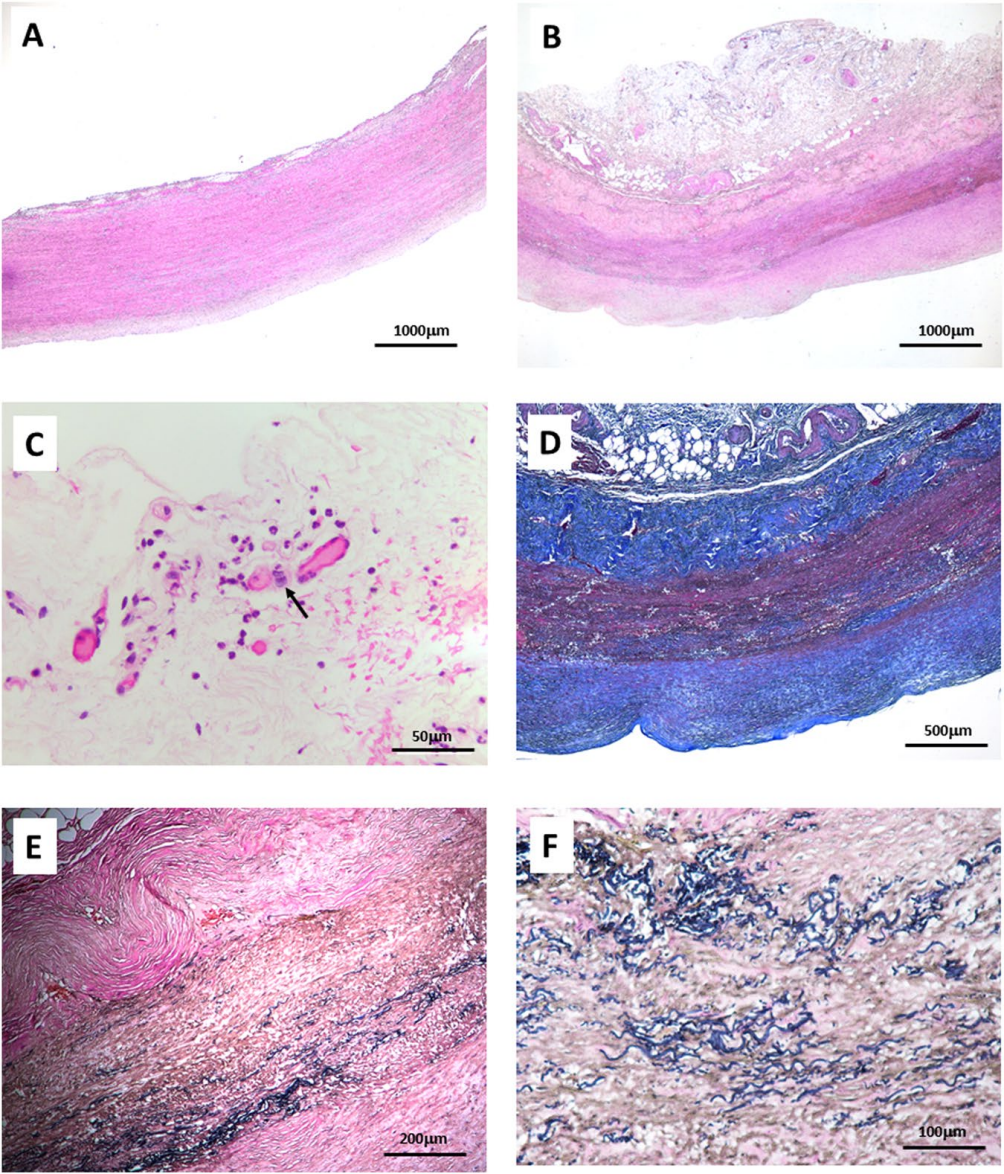
Histopathological features of aneurysms lesion in 26-year-old Takayasu arteritis patient with ascending aortic aneurysm. (**A**) Aortic wall tissue from a patient with no inflammatory lesion as a control(HE staining 20x), (**B**) Takayasu arteritis patient with aneurysm. The thichning of intima and media was observed (HE staining 20x), (**C**) Inflammatory cells infiltrating around the vasa vasorum in adventitia of the aortic wall was present, the arrow refers to the histocytes (HE staining 400x), (**D**) Masson stainning showed there was extensive collagen deposition in aortic wall tissue from Takayasu arteritis patient with aneurysm (40X), (**E**,**F**) Verhoeff-van Gieson stains highlighted extensive elastic fiber disorder, fracture and absence. (100x and 400x).

For histopathological studies, the aortic tissue from a 59-year-old male patient underwent aortic replacement surgery but had no inflammatory lesion was served as a control (Fig. 3A), and aortic tissue from a 26-year-old female TAK aneurysm patient who underwent the Bentall procedure (aortic root replacement) and partial aortic arch replacement was examined for histology. Hematoxylin & eosin staining revealed thickening of the vascular wall, inflammatory cells infiltrating the adventitia of the aortic wall, and lumen stenosis in the adventitial vasa vasorum in the aorta of TAK aneurysm. Masson stains showed extensive collagen deposition in aortic wall tissue from Takayasu arteritis patient with aneurysm. Furthermore, Verhoeff-van Gieson stains showed extensive elastic fiber disorder and fragmentation in the lesions of aneurysm (Fig. 3B–F).

### Analysis of the risk factors of aneurysms in takayasu arteritis patients

All baseline variables related to the formation of aneurysms were further analyzed using multivariate logistic regression analysis. The risk of aneurysms in TAK patients with elevated serum LDL-C levels was 5.8-fold higher than that of patients with normal LDL-C levels (p = 0.021, odd ratio [OR] = 5.767, 95% confidence interval [CI]: 1.302–25.543). The cutoff of serum TC and LDL-C levels for aneurysm were 4.60 mmol/l and 3.08 mmol/l, respectively. The risk of aneurysm was 4.2-fold higher in patients where the disease duration was over 60 months (p = 0.042, OR = 4.237, 95% CI: 1.055–17.023). The risk of the aneurysm in TAK patients with an elevated ESR was 2.9-fold higher than in patients with a normal ESR (p = 0.077, OR = 2.851, 95% CI: 0.891–9.115). The risk of aneurysm formation was increased 6.8-fold in patients with fever (p = 0.010, OR = 6.793, 95% CI 1.578–29.252) and 3.6-fold in patients with chest pain (p = 0.057, OR = 3.580, 95% CI: 0.964–13.299) compared with those without these symptoms (Table 4).

**Table 4:** Analysis of risk factors of aneurysm in Takayasu arteritis patients by the model of logistic regression.

Variables	OR	95% CI	P Value
Elevated LDL-C	5.767	1.302, 25.543	0.021
Disease duration > 60 months	4.237	1.055, 17.023	0.042
Elevated ESR	2.851	0.891, 9.115	0.077
Fever	6.793	1.578, 29.252	0.010
Chest pain	3.580	0.964, 13.299	0.057

Note: LDL-C, low-density lipoprotein cholesterol; ESR, erythrocyte sedimentation rate.

### Follow-up results in patients with aneurysms

Thirteen patients (61.9%) with 14 aneurysms were followed for a median duration of 18.3 months (range: 4–36 months). All patients were treated with immunosuppressive agents. Among of them, 1 patient underwent aortic replacement to remove the aneurysm and 1 developed a new aneurysm in the right subclavian artery. Among 13 of the aneurysms (12 patients), 46.2% (6/13) were enlarged, the median value of ratio of enlargement was 11.8%. 15.4% (2/13) showed no change, and 38.5% (5/13) showed reduced diameters and the median value of ratio of decrease was 3.2%. LDL-C levels were higher in TAK patients with progressive aneurysm than that in the non-progressive group (3.0 ± 1.4 mmol/l vs. 2.2 ± 0.4 mmol/l, respectively), but the difference was not significant because of the small number of samples (p = 0.191) (Table 5).

**Table 5:** Follow-up results of patients with aneurysm.

	With progressive (n = 5)	without progressive (n = 7)	P-value
Ratio of enlargement of aneurysm,%	11.8 (4.3, 69.2)	−3.2 (0.0, −8.6)	—
Corticosteroids, n(%)	2 (40.0)	5 (71.4)	—
Immunosuppressant, n(%)	5 (100.0)	7 (100.0)	—
Statin, n(%)	2 (40.0)	3 (42.9)	—
LDL-C (mmol/l)	3.0 ± 1.4	2.2 ± 0.4	0.191
ESR(mm/1 h)	8.0 (6.0, 21.5)	11.0 (7.0,17.0)	0.530
CRP (mg/l)	2.2 ± 1.2	3.1 ± 2.3	0.469

Note: LDL-C, low-density lipoprotein cholesterol; ESR, erythrocyte sedimentation rate; CRP, C-reactive protein.

## Discussion

In this study, we found that aneurysms most frequently occur in the thoracic aorta, and Numano type V was observed to be the most prevalent type. The serum TC and LDL-C levels were significantly higher in aneurysm patients than that in patients without aneurysm. Elevated LDL-C, ESR and disease duration of over 5 years were associated with the aneurysm formation in TAK patients. Immunosuppressive therapy can reduce aneurysms and patients with well-controlled aneurysms have relatively lower LDL-C level.

Recently, a prospective study has recognized that elevated LDL-C levels increase the risk for clinical AAA, and are also linked to asymptomatic AAA^3^. Genetic studies have revealed that increased LDL-C is associated with increased risk of AAA, suggesting that lowering LDL-C levels may offer an effective strategy for the prevention and management of AAA^4^. Previous studies have found that dyslipidemia is prevalent in patients with TAK, the rates were reported to be 46.7% in Brazil^10^, 36.3% in Korea^11^, 26.4% in Japan^12^, and 18.9% in France^13^. The incidence of dyslipidemia in our patients was 38.8%. In the present study, the risk of aortic aneurysm was 5.8-fold higher in patients with elevated LDL-C levels than in patients with normal LDL-C levels. The cutoff of serum TC and LDL-C levels for aneurysm were 4.60 mmol/l and 3.08 mmol/l, respectively. Estimate the threshold of serum TC or LDL-C levels in clinical practice that may be able to avoid the development of aortic aneurysm in TA patients.

Lipid metabolism disorders are related to the activity of TAK^14^. Inflammatory cytokines such as interleuken-6 and tumor necrosis factor-α can promote the LDL receptor-independent uptake of oxidized LDL. *In vitro*, these cytokines upregulate scavenger receptors in macrophages, driving the macrophages to transform into foam cells in patients with atherosclerosis^20^. Animal experiments confirmed that hypercholesterolemia promotes abdominal aortic aneurysms in C57BL/6 mice infused with angiotensin II receptors^21^. LDL, in its oxidized form (ox-LDL), induces endothelial dysfunction in humans^22^. *In vitro* studies have shown that the treatment of smooth muscle cells (SMCs) with ox-LDL leads to the formation of typical foam cells. This process is associated with a transition of SMCs to a synthetic phenotype^23^. Ox-LDL is accompanied by accumulated leucocytes and pro-inflammatory cytokines that stimulate the transcription of MMPs and contribute to extra-cellular matrix remodeling^24,25^. In this study, an increased risk of the formation of aneurysms was independent from atherosclerosis in TAK patients. The risk of aortic aneurysms was 5.8 times higher in patients with an elevated LDL-C level than in patients with a normal LDL-C level. We speculate that the serum LDL-C level exacerbated the formation of aneurysms in large vessel vasculitis.

TAK aneurysms may develop due to the long course of disease and progression of inflammation. The risk of aneurysm was 4.2-fold higher in patients with disease duration >5 years. Persistent inflammation of the aortic vessel wall is important risk factor for the development and progression of aortic aneurysms in TAK patients^26^. Inflammation in patients with TAK are thought to be mediated by activated monocytes, macrophages, and T-cells that synthetize pro-inflammatory cytokines, including TNF-α and IL-6^27^. Furthermore, increased cytokine levels within the lesions induce the production of MMPs in infiltrated mononuclear cells and/or smooth muscle cells, resulting in the destruction of elastic fibers in the arterial media in TAK^28^. Our study indicates that the location and mechanism of inflammatory aneurysms are significantly different from those of non-inflammatory aortic aneurysms^7^. Both dyslipidemia and vessel-wall inflammation are therefore important causes of aneurysm formation. With 18 months followed up, the diameter of aneurysm was reduced in 5 cases, this suggested immunosuppressive therapy can reduce aneurysms. Patients with well-controlled aneurysms have relatively lower LDL-C level, this may suggested control the level of LDL-C may be benefits to reverse the progress of aneurysm in TAK patients.

This study was limited by its retrospective design and the relatively small number of patients that were followed-up. Future cohort studies with larger numbers of patients and prospective studies that investigate statin interventions are needed to clarify the relationship between LDL-C and aneurysm formation in patients with TAK.

In conclusion, our study is the first to demonstrate that increased LDL-C level, ESR, and disease duration over 5 years may increase the risk of aneurysms in TAK patients. The results of this study suggest that the combination of elevated LDL-C and TAK is associated with the formation of aneurysms. Intensive evaluates serum LDL-C would help to reduce development of aneurysm in TAK patients.

### Declarations

Ethics approval and consent to participate: All subjects provided written informed consent. The study was conducted in accordance with the ethical principles of the Declaration of Helsinki and approved by the Clinical Research Ethics Board of Beijing Anzhen Hospital, Capital Medical University (Approval Number: 2018013X).

## Data Availability

No datasets were generated or analysed during the current study.
